# Computational methods for the integrative analysis of single-cell data

**DOI:** 10.1093/bib/bbaa042

**Published:** 2020-08-06

**Authors:** Mattia Forcato, Oriana Romano, Silvio Bicciato

**Affiliations:** 1 Molecular Biology and Bioinformatics at the University of Modena and Reggio Emilia. His research interests include the development and application of bioinformatics methods for the analysis of next-generation sequencing data; 2 Molecular Biology and Bioinformatics at the University of Modena and Reggio Emilia. Her research activities are mainly focused on the integrative analysis of transcriptional and epigenomic bulk and single-cell data; 3 Industrial Bioengineering at the University of Modena and Reggio Emilia. His research activity is the development and application of computational approaches for the analysis of multi -omics data

**Keywords:** bioinformatics, single cell genomics, data integration

## Abstract

Recent advances in single-cell technologies are providing exciting opportunities for dissecting tissue heterogeneity and investigating cell identity, fate and function. This is a pristine, exploding field that is flooding biologists with a new wave of data, each with its own specificities in terms of complexity and information content. The integrative analysis of genomic data, collected at different molecular layers from diverse cell populations, holds promise to address the full-scale complexity of biological systems. However, the combination of different single-cell genomic signals is computationally challenging, as these data are intrinsically heterogeneous for experimental, technical and biological reasons. Here, we describe the computational methods for the integrative analysis of single-cell genomic data, with a focus on the integration of single-cell RNA sequencing datasets and on the joint analysis of multimodal signals from individual cells.

## Introduction

As -omics data generated at single-cell resolution are rapidly increasing in throughput and quality, there is a great expectation that their analysis will provide fundamental insights into the regulatory interactions underlying complex phenotypes. However, the capacity to transform collections of single-cell signals into models of cellular functions depends on the efficacy to integrate multi-view information acquired from different biomolecular layers and cell populations. Multi -omics data entangle the cross-sectional hierarchy of biological phenomena and their integration allows accessing a systemic view of the molecular processes that is deeper than the one returned by the sum of the signals analyzed separately [1]. The integrative analysis of single-cell multimodal data provides a powerful framework to determine the correlations that occur among different molecular signals in the various cell types and to quantify the impact of these relationships in defining cell identities [2, 3]. Multimodal data from individual cells can be obtained simultaneously profiling multiple types of molecules within a single-cell or assembling signals collected from separate assays and distinct cells. In both cases, their integration requires computational approaches specifically designed to reconcile the immense heterogeneity observed across individual datasets. As for bulk experiments, multi-view single-cell data are high-dimensional and comprise many distinct yet interdependent signals, each with specific characteristics, dimensionality and noise. Across modalities, data are commonly collected in different genomic locations (genes, genomic regions), scales and formats (levels, states). Moreover, datasets vary widely in the number and type of profiled cells, of investigated biological samples (e.g. treatments, individuals) and for technical aspects (as sample processing, library preparation or sequencing depth). All these heterogeneities pose additional computational challenges to the application of methods previously developed for bulk experiments [4–7]. In recent years, a variety of approaches have been proposed to specifically address the integration of single-cell signals produced in various studies using multiple assays. Some methods deal with specific issues, such as the integration of multiple single-cell RNA sequencing (scRNA-seq) experiments or the classification of cell types in different studies, while some others address all aspects of single-cell integrative analysis, including the harmonization of multimodalities and the joint analysis of transcriptional and spatial expression. A majority of these methods build on machine learning techniques, owing to their ability to extract rules from multiple feature sets (multi-view learning; [8]), and obtain the integration either relating elements that have an attribute in common across diverse data types and datasets or combining features singularly extracted from each data view. Most approaches adopt a transformation-based integration, meaning that each data type is first transformed into an intermediate form (e.g. through dimensionality reduction) and then all transformations are merged to perform the downstream integrative analysis [1, 9, 10]. This strategy borrows elements from the standard analysis of individual scRNA-seq samples where the identification of a set of latent variables enables the algorithms to distinguish groups of cells sharing common genomic traits despite the intrinsic heterogeneity of the data. Under the assumption that, in a low-dimensional embedding, cells with the same identity or in the same state map close together although profiled with different assays, most approaches adopt computational techniques for the exploration of multidimensional data (such as multiple factor analysis, canonical correlation analysis or nonnegative matrix factorization) to match observations from different experiments and to identify functional correlations between data from single cells.

**Figure 1:**
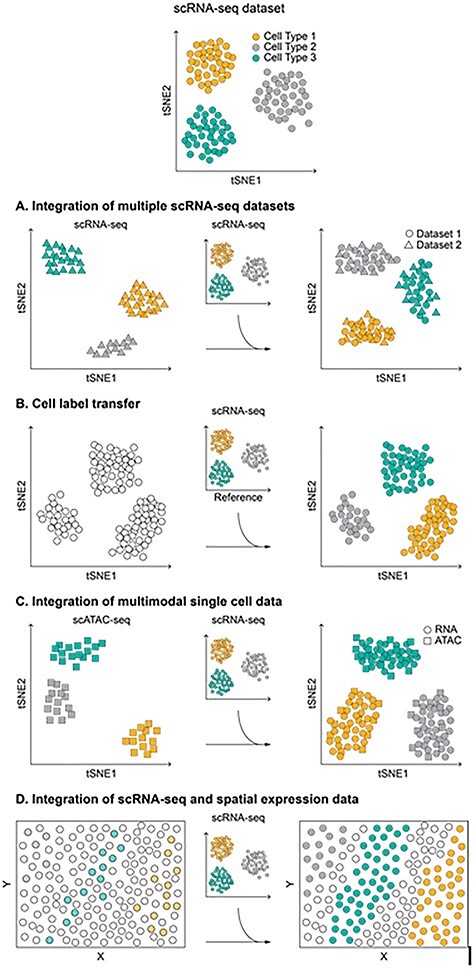
Schematic representation of the different types of single-cell data integration scenarios.

The goal of this survey is to present an overview of computational methods for the integrative analysis of single-cell data. We discuss the various approaches in the context of different types of single-cell data integration problems although this classification is largely instrumental to applications (Figure 1). Most of the tools reviewed here are also listed in the ‘integration’ and ‘classification’ categories of the scRNA tool database (scrna-tools.org; [11]).

**Table 1:** Methods for the integration of multiple scRNA-seq datasets

Tool name	Category	Algorithm	Output	Language	Availability	Reference
batchelor	Anchor-based	PCA and mutual nearest neighbor	Corrected gene expression matrix and integrated data in low-dimensional space	R	https://bioconductor.org/packages/devel/bioc/html/batchelor.html	[13]
SMNN	Anchor-based	PCA and mutual nearest neighbor	Corrected gene expression matrix	R	https://github.com/yycunc/SMNN	[17]
Scanorama	Anchor-based	SVD and mutual nearest neighbors matching	Corrected gene expression matrix and integrated data in low-dimensional space	Python	https://github.com/brianhie/scanorama	[18]
BEER	Anchor-based	PCA and mutual nearest neighbor	Selected PCA subspaces	R	https://github.com/jumphone/BEER	[19]
Seurat v3 (*IntegrateData*)	Anchor-based	CCA and mutual nearest neighbor	Corrected gene expression matrix and integrated data in low-dimensional space	R	https://github.com/satijalab/seurat/releases/tag/v3.0.0	[20]
Seurat v2	Other	CCA and dynamic time warping	Integrated data in low-dimensional space	R	https://github.com/satijalab/seurat/releases/tag/v2.0.0	[21]
Harmony (*RunHarmony*)	Anchor-based	PCA, fuzzy clustering and linear combination	Integrated data in low-dimensional space	R	https://github.com/immunogenomics/harmony	[22]
scNCA	Anchor-based	Context likelihood neighbors, NCA	Integrated data in low-dimensional space	R	https://github.com/gongx030/scNCA	[23]
scMerge	Anchor-based	Mutual nearest clusters (pseudo-replicates) and RUV-III factor analysis	Corrected gene expression matrix	R	https://github.com/SydneyBioX/scMerge	[24]
Conos (*buildGraph*)	Graph-based	Dimensionality reduction (PCA, CPCA, CCA, JNMF), mutual nearest neighbor, clustering	Corrected gene expression matrix and integrated data in low-dimensional space	R/C++	https://github.com/hms-dbmi/conos	[26]
BBKNN	Graph-based	PCA, KNN and UMAP	Integrated data in low-dimensional space	Python	https://github.com/Teichlab/bbknn	[27]
scPopCorn	Graph-based	PCA, PageRank and graph *k*-partitioning	Integrated data in low-dimensional space	Python	https://github.com/ncbi/scPopCorn	[28]
LIGER (*quantileAlignSNF*)	Combined anchor- and graph-based	iNMF and joint graph	Integrated data in low-dimensional space	R	https://github.com/MacoskoLab/liger	[30]
scAlign	Deep learning	Dimensionality reduction (PCA, CCA), neural networks	Corrected gene expression matrix and integrated data in low-dimensional space	R/Python	https://github.com/quon-titative-biology/scAlign	[31]

For the methods that perform multiple types of integrative analysis, the corresponding function for the integration of multiple scRNA-seq datasets is specified in the tool name. PCA, principal component analysis; SVD, singular value decomposition; CPCA, common PCA; JNMF, joint nonnegative matrix factorization; UMAP, Uniform Manifold Approximation and Projection.

## Integration of multiple scRNA-seq datasets

scRNA-seq is the most widely used technique for the analysis of gene expression levels within individual cells, and the number of scRNA-seq experiments has been constantly growing since its first application. With the rapid accumulation of scRNA-seq data, a major computational challenge is the integration of single-cell transcriptomes collected across different studies to increase the inference power (Figure 1A). As compared to single experiments, integrated scRNA-seq data account for a larger number of cells, thus facilitating the identification of both common and rare cell types and a finer definition of cell identities. Merging multiple scRNA-seq datasets requires to first remove all variations caused by batch effects (as differences in sample processing, library preparation or sequencing technology) or biological factors (e.g. treatments, individuals), which cause cells to cluster by sample, dataset or experiment, rather than by type [6, 12, 13]. When integrating bulk RNA-seq data, the batch effect is considered to be uniformly distributed across all samples and is removed using linear models that generate batch-corrected signals [14–16]. However, the assumption of uniformly distributed batch effects across cells and of conserved gene expression structure across datasets is not necessarily verified in scRNA-seq data. In addition, different scRNA-seq experiments generally capture cell types and populations that are unbalanced or only partially shared across experiments, making the integration further challenging. Therefore, computational strategies for the integration of bulk transcriptomes might result inadequate and several *ad hoc* procedures have been specifically developed to integrate multiple scRNA-seq experiments (Table 1). Before integration, essentially all methods preprocess each batch independently (through normalization and scaling) and perform feature selection to identify a set of highly variable genes (HVG) in any dataset, sufficiently large to guarantee the detection of even rare cell populations. The union of the HVGs that are expressed in all datasets constitutes the base for the subsequent integration of the different batches. For most methods, the integrative process comprises the identification of a low-dimensionality space, common to all datasets, where it is assumed that cells with the same identity or in the same state map close together although obtained from diverse experimental conditions and biological contexts. The identification of this space presupposes that the different batches share at least one population of cells. In the common low-dimensional space, distances among cells are used to estimate batch alignment vectors or to construct a joint-graph representation. In the first case, correction vectors are obtained from selected groups of cells in different batches that are used as anchors to align the various datasets. In the second, edges connecting cells in the joint graph are weighted according to cell distances and the batch alignment is obtained through community detection methods on the graph.

Methods for anchor-based alignment exploit a variety of unsupervised and supervised approaches to identify the anchoring cell pairs. For all these methods, the accuracy in determining the anchors is a critical factor for an effective data integration. The *mnnCorrect* and *fastMNN* functions of the *batchelor* R package determine anchors taking advantage of mutual nearest neighbor (MNN) cells, i.e. identifying pairs of cells that are mutually closest to each other across batches [13]. The difference between the expression profiles of MNN cells is then used to estimate the batch effect and compute the local correction vectors for each cell. The MNN concept is also used in SMNN [17], Scanorama [18], BEER [19] and Seurat v3 [20]. In SMNN, the detection of MNNs is restrained within cell populations matched on the base of user-defined marker genes. Scanorama generalizes the MNN approach implemented in *batchelor* to the simultaneous integration of multiple datasets, including time series. In BEER, MNN-based anchors are used to identify dimensions that account for latent batch effect and need to be discarded. Differently from these methods that apply principal component analysis or singular value decomposition for dimensionality reduction, Seurat v3 uses canonical correlation analysis (CCA) to identify a low-dimensional space where the correlation between the canonical variates is maximized. Anchors are defined as MNN cells in this reduced low-dimensional representation, filtered according to the original high-dimensional expression values and scored based on the shared overlap of mutual neighborhoods [20]. Instead of using anchors, a previous version of the Seurat suite (Seurat v2) integrates multiple scRNA-seq datasets in the CCA space aligning the canonical correlation vectors with the dynamic time warping algorithm, a nonlinear transformation also used for the comparison of single-cell trajectories [21]. In Harmony, anchors are determined in the principal component space as the dataset-specific centroids of clusters defined using a soft *k*-means algorithm [22]. Another approach similar to MNN correction has been proposed to integrate different scRNA-seq time series experiments. Specifically, the method named single-cell neighborhood component analysis (scNCA) determines cell context likelihood neighbors by comparing, within each time point, the distances between cells from two batches with a null model. Then, it applies a batch-specific linear transformation (similar to neighborhood component analysis, NCA) that maximizes the closeness of cells with high context likelihood, preserving the local trajectories [23]. The anchor concept underlies also the approach developed in scMerge [24]. Here, cells that form the core of mutual nearest clusters across datasets are considered as pseudo-replicates. Pseudo-replicates are then used as replicates in an extended version of the RUV-III normalization strategy [25] to estimate the unwanted variation of stably expressed genes (negative control) and to return a single batch-corrected expression matrix that can be used for downstream analyses.

Conos [26], BBKNN [27] and scPopCorn [28] implement joint graph-based approaches. In Conos (Clustering on network of samples), the integration of different datasets is obtained generating a weighted graph representation where shared populations are identified by community detection methods. The graph is constructed considering as nodes the cells of all datasets connected by both inter- and intra-dataset edges. Weights of the inter-dataset edges are calculated given a neighbor mapping strategy in a rotation space (e.g. MNN in common principal components space). The intra-dataset edges are weighted according to distances calculated, within each batch, in the space of the top principal components and downscaled to reduce their contribution in the joint graph. The graph is then clustered using Walktrap, Louvain or Leiden community detection methods to identify joint clusters connecting cell populations across datasets. BBKNN (batch balanced *k*-nearest neighbors) returns a joint graph where inter- and intra-dataset edges are computed independently on each batch using KNN and edge weights are assigned according to the Uniform Manifold Approximation and Projection strategy [29]. In scPopCorn, the inter-dataset edges are defined by a cosine similarity matrix computed on the intersection of the HVGs. Instead, the intra-dataset edges are obtained from a co-membership propensity graph constructed within each batch applying an adaptation of the PageRank algorithm to the cosine similarity matrix. The resulting joint graph is processed using a *k*-partitioning method to define connected components that represent subpopulations shared by the different datasets.

Linked inference of genomic experimental relationships (LIGER) exploits concepts of both the anchor- and graph-based approaches [30]. Here, integrative non-negative matrix factorization (iNMF) is first applied to determine a low-dimensional space in which each cell is defined by factors specific to each dataset and by factors shared among batches. A shared factor neighborhood graph that connects cells with similar factor loading across batches is then constructed in the factor space and analyzed with Louvain community detection to identify joint clusters across datasets. The final data alignment is obtained correcting the factor loadings within each joint cluster through the match of their quantiles across datasets.

Finally, in scAlign, data integration is obtained using a deep learning approach based on neural networks [31]. Specifically, first a neural network encoder is trained to learn a low-dimensional embedding where cells in the same state overlap across batches and then a decoder network is used to recreate the gene expression signals of each input dataset projecting cells from the alignment space back to the input space.

Depending on the method, the integrative analysis returns data corrected in the low-dimensional space, corrected expression signals or both. However, the use of corrected expression values to perform downstream analyses, other than visualization, must be carefully evaluated since dataset alignment may introduce artificial expression difference that affects the identification of cell type and the interpretation of results [32].

### Cell label transfer from reference scRNA-seq datasets

The integration of multiple scRNA-seq datasets can also be used to annotate cell populations. The annotation of cell types is a typical step in the analysis of scRNA-seq data and is commonly addressed first clustering cells based on their gene expression profiles and then annotating the clusters using cell type-specific markers. Although effective, this process is mostly manual, requires prior knowledge of genes and signatures that specifically mark different cell types and suffers the complexity of acquiring ground truth from experimentally purified subpopulations [33,34]. An alternative approach for cell annotation is to take advantage of well-annotated scRNA-seq atlases and automatically transfer cell type classifications from a reference to a query dataset (Figure 1B; Table 2). Some of the tools previously described for the integration of scRNA-seq data contain routines for the automatic transfer of cell labels [20, 26]. In particular, Seurat v3 identifies the anchors between reference and query sets and uses the anchor weights to predict labels of the query cells [20]. Conos, once integrated a reference and a query scRNA-seq dataset in a joint graph, can propagate cell labels between graph vertices, thus annotating the unlabeled cells of the query set [26]. Notably, both Seurat v3 and Conos can transfer labels across multiple modalities, as for instance annotate cells from a scATAC-seq experiment using transcriptional profiles as reference. A similar approach is exploited by cellHarmony that, although not directly integrating reference and query data, first defines cell communities in each dataset, then align individual cells within matched communities and finally assign to each query cell the label of the closest matching reference cell [35].

**Table 2:** Methods for the automatic cell label transfer from reference scRNA-seq datasets

Name	Category	Algorithm	Language	Availability	Reference
Seurat v3 (*TransferData*)	Integration-based	Weighted vote classifier based on integration anchors	R	https://github.com/satijalab/seurat/releases/tag/v3.0.0	[20]
Conos (*propagateLabels*)	Integration-based	Label propagation within joint graph	R/C++	https://github.com/hms-dbmi/conos	[26]
cellHarmony	Integration-based	Closest neighbor within community clusters	Python	https://github.com/AltAnalyze/cellHarmony-Align	[35]
scmap	Classification model	Similarity measures	R	http://bioconductor.org/packages/release/bioc/html/scmap.html	[36]
scID	Classification model	Linear discriminant analysis and mixture Gaussian model	R	https://batadalab.github.io/scID/	[37]
Moana	Classification model	Support vector machines with linear kernel	Python	https://github.com/yanailab/moana	[38]
scPred	Classification model	Support vector machines with radial kernel	R	https://github.com/powellgenomicslab/scPred	[39]
SuperCT	Classification model	Artificial neural network	Keras API	https://sct.lifegen.com/	[40]
ACTINN	Classification model	Artificial Neural Network	Python	https://github.com/mafeiyang/ACTINN	[41]
LAmbDA	Classification model	Artificial Neural Network	Python	github.com/tsteelejohnson91/LAmbDA	[42]
CaSTLe	Classification model	Boosted regression trees	R	https://github.com/yuvallb/CaSTLe	[43]
singleCellNet	Classification model	Random Forest	R	https://github.com/pcahan1/singleCellNet/	[44]
CHETAH	Cell type hierarchy	Correlation to reference tree	R	https://github.com/jdekanter/CHETAH	[45]
Garnett	Cell type hierarchy	Elastic-net regression on reference tree	R	https://cole-trapnell-lab.github.io/garnett/	[46]
OnClass	Cell type hierarchy	Cell ontology projection into a low-dimensional space	Python	https://github.com/wangshenguiuc/OnClass/	[47]

For the methods that perform multiple types of integrative analysis, the corresponding function for the automatic cell label transfer is specified in the tool name.

Several other methods formulate cell label transfer as a classification problem and construct a classification model on the reference dataset to predict the query cell labels. These tools implement a variety of classification strategies ranging from the quantification of the similarity between clusters in the reference and in the query to the adoption of supervised machine learning algorithms. In scmap, each cell of the query dataset is classified based on its similarity with each reference cluster centroid (scmap-cluster) or based on the annotation of its KNN in the reference (scmap-cell) [36]. Cell types are predicted through cluster comparison also in scID where a Gaussian mixture model is used to assign the query cells to the reference cluster with the highest likelihood, given a score defined by cluster-specific genes identified in the reference dataset [37]. Similarly, in Moana, the cell types of the query dataset are predicted using linear support vector machine (SVM) models trained on the clusters identified in the two-dimensional PC space of the reference set [38]. In scPred [39], a SVM model is trained using the most informative principal components of the training set. SuperCT [40], ACTINN [41] and LAmbDA [42] exploit the knowledge encoding of artificial neural networks to predict unknown cell types from binarized or digital transcriptional data, and CaSTLe [43] and singleCellNet [44] implement an ensemble of boosted regression trees and a Random Forest classifier, respectively. Finally, some methods transform the reference set into a hierarchy of cell types that is further used to predict the query labels. The hierarchy can be constructed in an unsupervised manner directly from the reference data as in CHETAH [45] or defined using a priori knowledge as in Garnett [46] and OnClass [47]. In general, it is worth noting that the robustness of cell classification relies not only on the algorithmic choice but also on the quality and detail of the reference annotation. Specifically, as reported by two recent comparison studies, the various methods are diversely affected by the size and cell type proportions of the reference [33] and tend to underperform in the presence of overlapping and overly detailed annotations [34].

## Integrative analysis of multimodal single-cell data

Multimodal single-cell data can be obtained directly using multimodal technologies, which simultaneously profile multiple types of molecules within a single cell, or through computational methods that integrate, into a single-cell multi-omics dataset, signals collected from separate assays (Figure 1C). Technologies for the simultaneous profiling of single-cell multi-omics have been extensively reviewed in [2, 3, 6, 48, 49]. Here, we will discuss only computational approaches for the integrative analysis of multimodal data (Table 3). The integration of multiple modalities from a single experiment benefits from the fact that all measurements are from the same cells. This obviates the need to reconcile cell identity across modalities and restricts the analysis to the implementation of procedures to correlate complementary information from multiple sources. The correlation of different modalities commonly starts summarizing the signals of one modality relatively to the genomic entities assayed by the other before testing for association. This approach is clearly exemplified in two studies that integrated data from the parallel single-cell profiling of DNA methylation and gene expression [50,51]. First, average binarized methylation rates of genomic regions (as gene bodies, promoters and enhancers) have been assigned to the closest gene, considering windows of 10 kb upstream and downstream the gene transcription start and end sites. Then, weighted Pearson correlation coefficients [50, 51] and multi-omics factor analysis (MOFA), an adaptation of group factor analysis to multimodal -omics data [52], have been used to identify coordinated changes and heterogeneous associations between methylation states and transcriptional profiles in individual cells. Recently, MOFA has been generalized in the MOFA+ approach to support large-scale datasets and to handle multiple batches among the modalities [53].

**Table 3:** Methods for the integration of single-cell data from different modalities

Tool name	Category	Algorithm	Language	Availability	Reference
scMT-seq analysis	Integration of multimodal data from the same cells	Weighted Pearson correlation	R	https://github.com/PMBio/scMT-seq	[50]
MOFA+	Integration of multimodal data from the same cells	Bayesian factor analysis framework	R/Python	https://github.com/bioFAM/MOFA2	[52, 53]
ATAC_RNA_FIT	Model from bulk data	Linear model from bulk data and Pearson correlation	Matlab	https://ars.els-cdn.com/content/image/1-s2.0-S009286741830446X-mmc4.zip	[54]
CoupledNMF	Model from bulk data	Linear model from bulk data and NMF	Matlab	http://web.stanford.edu/~zduren/CoupledNMF/	[55]
Seurat v3 (*TransferData*)	Matching on the same genomic entities	Canonical correlation analysis	R	https://github.com/satijalab/seurat/releases/tag/v3.0.0	[20]
Conos (*buildGraph*)	Matching on the same genomic entities	Dimensionality reduction, mutual nearest neighbor and clustering	R/C++	https://github.com/hms-dbmi/conos	[26]
LIGER (*quantileAlignSNF*)	Matching on the same genomic entities	iNMF and joint graph	R	https://github.com/MacoskoLab/liger	[30]
clonealign	Matching on the same genomic entities	Inference model of gene expression on copy number	R	https://github.com/kieranrcampbell/clonealign	[57]
SOMatic	Matching on the same genomic entities	Self-organizing maps	R/C++	https://github.com/csjansen/SOMatic	[58]
MATCHER	Matching on the pseudotime values	Gaussian process latent variable model (GPLVM) and time warping	Python	https://github.com/jw156605/MATCHER	[59]

For the methods that perform multiple types of integrative analysis, the corresponding function for the integration of multimodal single-cell data is specified in the tool name.

Conversely, the integration of different types of data that are not measured on the same cell not only requires to aggregate signals measured by distinct technologies but also to align data collected from different cells. The analysis builds on the assumption that cells of the same type or in the same state share a set of correlated features. These features guide both the combination of signals from the different modalities and the matching of profiles across datasets. Signal pairing and dataset integration can be obtained either exploiting models formulated on bulk data or through algorithms that directly align multiple datasets based on the sole single-cell data. The first approach has been used to integrate scRNA-seq and scATAC-seq data in [54, 55]. Here, linear models to estimate gene expression levels from chromatin accessibility of gene regulatory elements have been first constructed using paired RNA- and ATAC-seq bulk data and then applied to guide the matching of single-cell profiles. In [54], scRNA-seq profiles are assigned to scATAC-seq cells based on the correlation between the gene expression levels measured by scRNA-seq and those inferred by the model for each scATAC-seq cell. In CoupledNMF, the linear model is used to combine the non-negative factorization of the scATAC-seq and scRNA-seq matrices through a coupling term that imposes the matching between features of one dataset and linear transformed features of the other [55].

Multimodal integration can be obtained from single-cell data alone using some of the methods previously described to align scRNA-seq datasets from different experiments, provided that the signals from the two modalities are previously matched on the same genomic entities. In particular, Seurat v3 [20] and Conos [26] have been applied to the integration of scRNA-seq and chromatin accessibility data, after the transformation of scATAC-seq accessibility peaks into gene-centered scores of accessibility-based activity. This can be obtained either summing scATAC-seq signals intersecting the gene body and an upstream region of given length (e.g. 2 kb) or combining signals of proximal and distal *cis*-regulatory regions predicted on the base of co-accessibility scores, as done by Cicero [56]. Similarly, LIGER has been used to integrate gene expression levels and DNA methylation after the annotation of the methylated regions according to the nearest gene [30].

Approaches specifically designed for the integration of single-cell multimodal data have been implemented in clonealign [57], SOMatic [58] and MATCHER [59]. In clonealign, a cell profiled with scRNA-seq is assigned to a specific clone assayed with scDNA-seq based on the probability that the observed gene expression levels are consistent with the copy number profiles of the clone. SOMatic uses self-organizing maps (SOM) to integrate scRNA-seq and scATAC-seq data. Briefly, distinct SOM are constructed for each data type and aggregated into meta-clusters using a *k*-means algorithm. Then, meta-clusters are used to link transcriptional and chromatin accessibility data based on the overlap between genes and regions with scATAC-seq peaks. In the case it is possible to assume an intrinsic order of cells as, for instance, in differentiation or reprogramming experiments, the integration of multimodal single-cell data can be achieved through the identification of a one-dimensional space, encoding the common developmental process, where equivalent cells, although assayed by different modalities, are aligned. The approach implemented in MATCHER (Manifold Alignment to CHaracterize Experimental Relationships) determines this one-dimensional alignment space first inferring, separately for each dataset, a single latent variable (pseudotime) with a Gaussian process latent variable model and then aligning the pseudotime values from the different modalities through a warping function [59]. In the alignment space, signals from the different modalities (e.g. gene expression, DNA methylation, chromatin accessibility and histone modifications) are directly comparable, allowing to investigate the correlation between several features across individual cells.

**Table 4:** Methods for the joint analysis of scRNA-seq and spatial data

Tool name	Category	Algorithm	Language	Availability	Reference
spatial_mapping	Spatial reference map of landmark genes	Correspondence score based on logistic function	R	https://github.com/jbogp/nbt_spatial_backmapping	[60]
Seurat v1.1	Spatial reference map of landmark genes	LASSO, Gaussian models and multivariate normal models	R	https://github.com/satijalab/seurat/releases	[61]
DistMap	Spatial reference map of landmark genes	Matthews correlation coefficients and distribute mapping scores	R	https://github.com/rajewsky-lab/distmap	[62]
Algorithm for zonation profiles	Spatial reference map of landmark genes	Posterior probability matrix	Matlab	upon request	[63]
novoSpaRc	*De novo* reconstruction for tissue with intrinsic shape	Entropically regularized optimal transport	Python	https://github.com/rajewsky-lab/novosparc	[64]
SCHEMA	Conventional integration	Quadratic programming to find a single embedding maximizing distance correlation	Python	https://github.com/rs239/schema	[66]
Seurat v3 (*TransferData*)	Conventional integration	Anchors from canonical correlation analysis	R	https://github.com/satijalab/seurat/releases/tag/v3.0.0	[20]
LIGER (*quantileAlignSNF*)	Conventional integration	iNMF and joint graph	R	https://github.com/MacoskoLab/liger	[30]
Harmony (*RunHarmony*)	Conventional integration	PCA, fuzzy clustering and linear combination	R	https://github.com/immunogenomics/harmony	[22]

For the methods that perform multiple types of integrative analysis, the corresponding function for the joint analysis of scRNA-seq and spatial data is specified in the tool name.

## Integrative analysis of scRNA-seq and spatial expression data

The spatial localization of cells within tissues plays a crucial role in determining their functions and states. Thus, assessing the topological arrangement of single cells within their multicellular context is of paramount importance to fully characterize cell identity and behavior. Unfortunately, information about the spatial organization in the original tissue is not entirely conserved in single-cell expression data since tissues are commonly dissociated before cell isolation and transcriptional profiling. This experimental limitation can be overcome through the integration of gene and spatial expression data, and consequently, several computational approaches have been designed to infer the topological location in the original tissue of cells profiled by scRNA-seq (Figure 1D; Table 4). Most of these methods require, in addition to single-cell expression profiles, the availability of a spatial reference map consisting of measurements, in the intact tissue, of the spatial expression for landmark genes [60–63]. The key step of these approaches is the formulation of a statistical model to infer, given the values in the scRNA-seq matrix and the spatial expression of the landmark genes, the probability that a cell originated from any of the locations probed in the reference map (e.g. voxel, bin or layer). In model construction, the spatial expression data are either inputted as continuous values or transformed into binarized on/off states. The various methods implement different computational strategies to formulate the inference models. In [60], binarized vectors, accounting for the specificity of each gene’s expression in a cell of the scRNA-seq dataset and in a voxel of the reference atlas, are compared to define a cell-voxel correspondence score. DistMap [62] calculates, for each cell, a confusion matrix summarizing the agreement between its binarized gene expression and the gene expression patterns of all positional bins of the reference atlas. The Matthews correlation coefficient, a measure of the quality of the agreement, is used to assign a cell-bin score and perform positional assignment of each cell. Other methods formulate complete probabilistic inference framework building models of gene expression for the measured landmark genes in each bin or layer to infer the cell’s spatial location [61, 63].

Interestingly, it has been recently proposed a method, named novoSpaRc, that reconstructs the spatial organization of cell from scRNA-seq data using either a very limited reference atlas or no spatial information at all [64]. novoSpaRc can map cells on tissues with intrinsic shapes either with the support of spatial data of a small number of marker genes or starting from the sole transcriptional signals (*de novo* reconstruction). The reconstruction of the spatial cellular arrangement is formulated as a generalized optimal-transport problem that is resolved using an iterative algorithm under the assumption that physically contiguous cells tend to share overall similar transcriptional profiles.

With the advent of technologies for the high-throughput profiling of spatial expression, the integrative analysis of transcriptional profiles and spatial information can also be addressed in terms of conventional multimodal integration. In SCHEMA, the integration of expression and localization data, simultaneously measured with Slide-seq [65], is obtained through the identification of an affine transformation of the gene expression matrix constrained on the correlation between top NMF factors of the transcriptional data with the kernel-derived spatial density scores and the categorical labels of cell types [66]. Seurat v3 [20] and LIGER [30] have been applied to combine different scRNA-seq datasets with high-throughput single-cell transcriptional profiles measured *in situ* using the STARmap technique [67]. As for the integration of scRNA-seq with other modalities, the two methods exploit anchors determined through CCA and factors identified by iNMF, respectively, to transfer expression levels and cell types from the RNA sequencing matrix to the spatial transcription data. This information transfer enables a finer functional classification of spatially resolved cells and the prediction of spatial transcriptional signals for genes that were not probed by STARmap. Finally, also Harmony [22] has been applied to the integration of scRNA-seq with spatial expression data from multiplexed error robust fluorescence *in situ* hybridization (MERFISH).

## Concluding remarks

Single-cell technologies are boosting the capacity to resolve cellular and tissue complexity at an unprecedented pace and to move towards more systemic landscapes, where the study of the cell components is elevated to higher hierarchies, as genomic domains, regulatory modules and networks of interactions. An essential prerequisite to advance in this endeavor is the availability of computational approaches to simultaneously analyze the different, interconnected layers of information encoded by multimodal signals at the single-cell resolution. Although sophisticated and precise, current methods for the integrative analysis of bulk multi -omics data suffer the heterogeneous nature of single-cell data where confounding factors, intrinsic of each modality, hamper their effective longitudinal combination. To overcome this limitation, in the recent years, major efforts have been committed in the development of computational strategies for the integration of single-cell data obtained from different cell populations, in different studies and using different assays. The majority of these methods exploit algorithms commonly adopted in multi-view machine learning and build on sophisticated techniques for dimensionality reduction, pattern recognition, graph analysis, maximum likelihood estimation and statistical modeling. In most applications, the integrative analysis is approached in an unsupervised manner to identify cell types shared by different datasets or previously unknown correlations between modalities. In some other cases, the integration is conducted supervising the transfer of information from one dataset to another (as in cell label transfer from well-annotated atlases) or across different layers (as in the joint analysis of transcriptional and spatial expression). Tools are mostly implemented in Python, R, C++ and Matlab taking advantage of packages that efficiently implement machine learning and statistical models for the analysis of large, multidimensional data. However, considering the pace of data production, a primary issue will be the development of methods able to integrate larger and larger compendia of high-resolution datasets using reasonable computational resources and processing time. Another priority will be a systematic evaluation of the robustness of the different methods in producing biologically sound results from the analysis of multiple types of data and the definition of guidelines to efficiently integrate different data sources [68]. This benchmarking requires gold-standard experiments and standardized performance metrics to assess the quality of the integration approaches and to rationally tune the parameters of the various algorithms, thus limiting the heuristics inherent in any method. Although complicated by difficulties in simulating realistic multi-omics single-cell data [69, 70] and by the unsupervised nature of most analyses, benchmarking frameworks and performance tests have been proposed for the computational tools used in the integrative analysis of multiple scRNA-seq datasets [70–72] and in cell type classification [33, 34]. The performance and usability of the various tools for scRNA-seq batch integration have been critically evaluated in a recent benchmarking paper by Tran and colleagues [73]. This comparison indicates that there is no method that can be considered the gold standard under all scenarios. Nonetheless, Harmony, LIGER and Seurat v3 stand, overall, as the most effective methods for batch integration, while scMerge is the recommended tool to quantify differential expression on the corrected data. It is advisable that similar comparison approaches are extended to all types of single-cell data integrations. We believe that, in the years to come, the integrative analysis of multimodal single-cell data will have to face major methodological and applicative challenges but will also be instrumental to characterize novel exciting aspects of the fundamental mechanisms that regulate cell identity and tissue complexity.

**Key Points**

- We review computational methods for the integrative analysis of single-cell data obtained from different experiments and modalities.
- The integration of multiple scRNA-seq experiments comprises the identification of a common low-dimensionality space where cells with the same identity or in the same state map close together even if obtained from diverse experimental conditions and biological contexts.
- The integration of multiple scRNA-seq datasets can guide the annotation of cell populations.
- The integration of different types of single-cell data that are not measured on the same cell requires to aggregate signals measured by distinct technologies and to align cells from different experiments.
- The integration of gene and spatial expression allows inferring the topological location in the original tissue of cells profiled by scRNA-seq.

## Notes

**Mattia Forcato** is an assistant professor in Molecular Biology and Bioinformatics at the University of Modena and Reggio Emilia. His research interests include the development and application of bioinformatics methods for the analysis of next-generation sequencing data.

**Oriana Romano** is a post-doctoral associate in Molecular Biology and Bioinformatics at the University of Modena and Reggio Emilia. Her research activities are mainly focused on the integrative analysis of transcriptional and epigenomic bulk and single-cell data.

**Silvio Bicciato** is a professor of Industrial Bioengineering at the University of Modena and Reggio Emilia. His research activity is the development and application of computational approaches for the analysis of multi -omics data.
